# New potential *Plasmodium brasilianum* hosts: tamarin and marmoset monkeys (family Callitrichidae)

**DOI:** 10.1186/s12936-017-1724-0

**Published:** 2017-02-10

**Authors:** Denise A. M. Alvarenga, Anielle Pina-Costa, Cesare Bianco, Silvia B. Moreira, Patricia Brasil, Alcides Pissinatti, Claudio T. Daniel-Ribeiro, Cristiana F. A. Brito

**Affiliations:** 1 Laboratório de Malária, Centro de Pesquisa René Rachou (CPqRR), Fundação Oswaldo Cruz (Fiocruz), Belo Horizonte, MG Brazil; 2 Laboratório de Doenças Febris Agudas, Instituto Nacional de Infectologia (INI), Fiocruz, Rio de Janeiro, RJ Brazil; 3 Centro de Pesquisa, Diagnóstico e Treinamento em Malária (CPD-Mal), Fiocruz, Rio de Janeiro, RJ Brazil; 4 Laboratório de Pesquisa em Malária, Instituto Oswaldo Cruz (IOC), Fiocruz, Rio de Janeiro, RJ Brazil; 5 Centro de Primatologia do Rio de Janeiro (CPRJ/INEA), Rio de Janeiro, RJ Brazil; 6 Centro Universitário Serra dos Órgãos (UNIFESO), Rio de Janeiro, RJ Brazil

**Keywords:** Malaria, *Plasmodium brasilianum*, Callitrichidae, *Callithrix*, *Mico*, *Leontopithecus*, Atlantic forest

## Abstract

**Background:**

Non-human primates (NHPs) as a source for *Plasmodium* infections in humans are a challenge for malaria elimination. In Brazil, two species of *Plasmodium* have been described infecting NHPs, *Plasmodium brasilianum* and *Plasmodium simium*. Both species are infective to man. *Plasmodium brasilianum* resembles morphologically, genetically and immunologically the human quartan *Plasmodium malariae*. *Plasmodium brasilianum* naturally infects species of non-human primates from all New World monkey families from a large geographic area. In the family Callitrichidae only the genus *Saguinus* has been described infected so far. The present study describes the natural infection of *P. brasilianum* in tamarins and marmosets of the genera *Callithrix*, *Mico* and *Leontopithecus* in the Atlantic forest.

**Methods:**

One hundred and twenty-two NHPs of the family Callitrichidae housed in the Primate Centre of Rio de Janeiro (CPRJ) were sampled in June 2015, and January and July 2016. The CPRJ is located in the Atlantic forest in the Guapimirim municipality, in the Rio de Janeiro state, where human autochthonous cases of malaria have been reported. The samples were screened for the presence of *Plasmodium* using optical microscopy and nested PCR for detection of 18S small subunit rRNA gene. The amplicon was sequenced to confirm the molecular diagnosis.

**Results:**

The frequency of *Plasmodium* infections detected by nested PCR in New World monkeys of the family Callitrichidae was 6.6%. For the first time, Callitrichidae primates of genera *Callithrix*, *Mico* and *Leontopithecus* were found naturally infected with *P. brasilianum*. Infection was confirmed by sequencing a small fragment of 18S rRNA gene, although no parasites were detected in blood smears.

**Conclusions:**

The reported *P. brasilianum* infection in NHP species maintained in captivity suggests that infection can be favoured by the presence of vectors and the proximity between known (and unknown) hosts of malaria. Thus, the list of potential malaria reservoirs needs to be further explored.

**Electronic supplementary material:**

The online version of this article (doi:10.1186/s12936-017-1724-0) contains supplementary material, which is available to authorized users.

## Background

Many efforts are currently being undertaken towards control of malaria around the world. However, despite a 37% reduction in global malaria incidence and 60% in deaths in the last 15 years (2000–2015), the disease still remains an important public health problem in many tropical and sub-tropical countries [1]. It is a mosquito-borne disease of humans and other vertebrates caused by parasites of the genus *Plasmodium*. In the context of the malaria Eradication Research Agenda (malERA), non-human primates (NHPs) have increased their importance as source for *Plasmodium* infections in humans [2–4]. Therefore, there is now a consensus that some species of *Plasmodium* that typically infect NHPs, such as *Plasmodium cynomolgi* and *Plasmodium knowlesi* in Asia and *Plasmodium brasilianum* and *Plasmodium simium* in the Americas, may under special conditions infect humans [4–7].

*Plasmodium brasilianum* and *P. simium* are considered to be morphologically, genetically and immunologically highly similar to the human malaria parasites *Plasmodium malariae* and *Plasmodium vivax*, respectively [4, 8–13]. *Plasmodium brasilianum* was first described in *Cacajao calvus* (bald-uakari) from the Amazonas state in Brazil. This parasite is widely spread throughout Central and South America; it has been found in the Amazon forest of Panama, Venezuela, Colombia, French Guiana, Peru and Brazil, as well as in the Atlantic forest in the Brazilian Southern and Southeastern regions [4, 11, 14–19]. It has a remarkable plasticity, being able to infect a large number of species belonging to all families of New World monkeys, i.e., families Aotidae, Atelidae, Callitrichidae, Cebidae and Pitheciidae [11, 19–25]. Although the family Callitrichidae has six genera: *Callithrix, Cebuella, Leontopithecus, Mico, Saguinus* and *Callibella* [26, 27], *P. brasilianum* has been reported naturally infecting only the genus *Saguinus* (*S. geoffroyi*, *S. midas* and *S. niger)* [11, 21, 28].

Until the 1950s, malaria was a nationwide problem in Brazil. After successive and often successful campaigns, the disease was controlled in the majority of the national territory, remaining endemic only in the Amazon basin (Northern Brazil) [29, 30]. However, autochthonous cases of the disease have been described in the extra-Amazon region, most of them in the Southern and Southeastern Atlantic forest. Between 2006 and 2014 sixty-one malaria autochthonous cases were reported in Rio de Janeiro (RJ) state in the Southeastern with an average of 6.8 cases per year [31]. Between January 2015 and July 2016, 49 patients diagnosed with malaria reported the infection location as the Atlantic forest in RJ state [32]. Many studies suggest that the maintenance of autochthonous malaria in the Atlantic forest may involves the presence of infected NHPs acting as reservoirs of the disease. In these environments, malaria transmission would be carried out by the bromeliad mosquito *Anopheles (Kerteszia) cruzii,* the natural vector of both human and NHP malaria in the Atlantic forest [11]. Therefore, malaria in these mountain valleys may be a zoonosis [23, 33–35].

The report of autochthonous malaria cases in the RJ state has motivated the search for infected NHPs in its Atlantic forest region [35, 36]. The present study describes for the first time the natural infection of *P. brasilianum* in the genera *Callithrix*, *Mico* and *Leontopithecus* (family Callitrichidae) using molecular approaches.

## Methods

### Study area, ethics and monkey blood samples

**Fig. 1:**
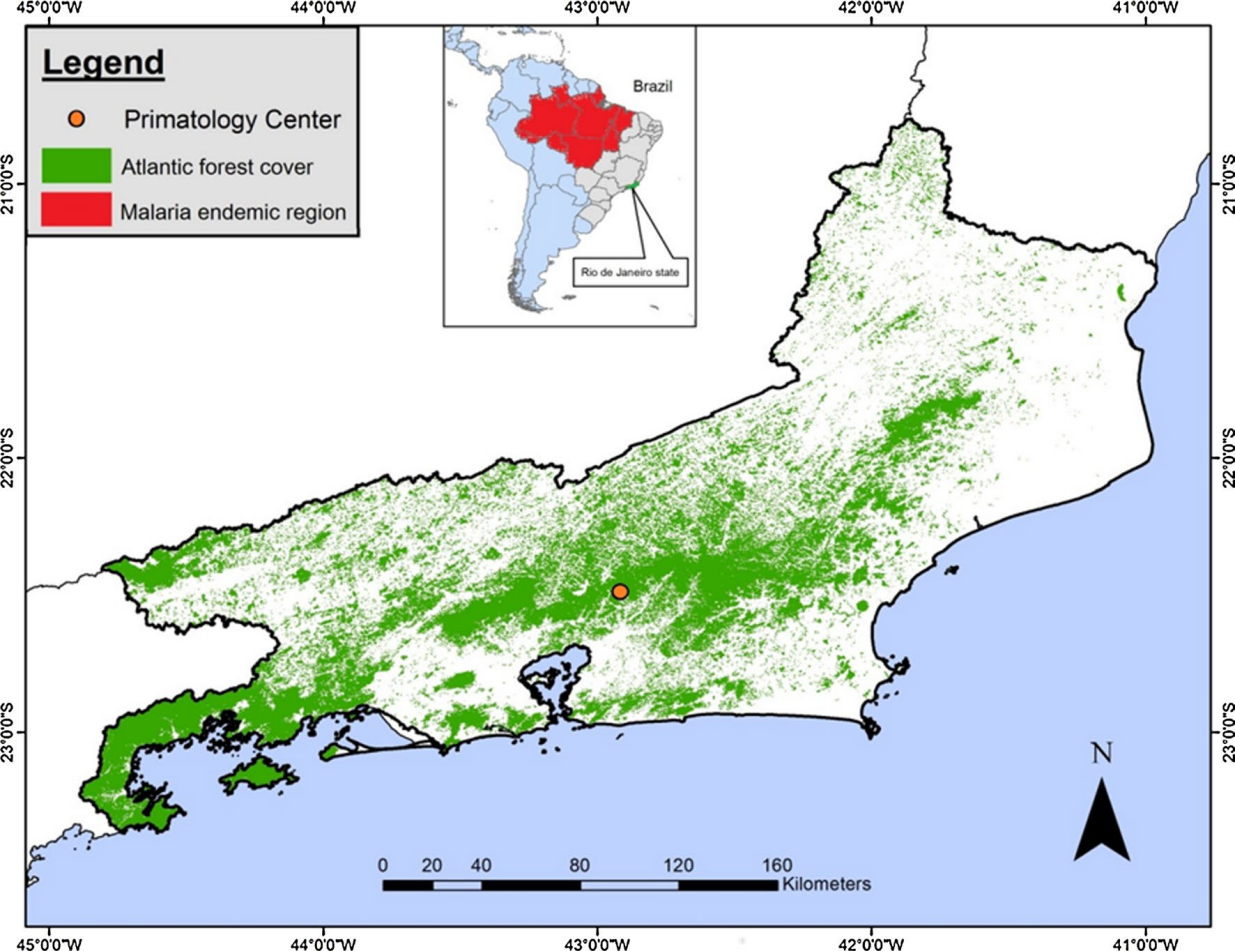
Map of Rio de Janeiro state showing the localization of the Primate Centre of Rio de Janeiro (CPRJ) (*Orange dot*), in Guapimirim Municipality, in the Atlantic Forest (*green*). In the detail, the map of Brazil (in South America) highlights the Amazonian (malaria endemic) area (*in red*)

NHPs from the Primate Centre of Rio de Janeiro (CPRJ, according to its initials in Portuguese) were studied in this survey. The CPRJ, part of the Brazilian Institute of Environment and renewable natural resources (Register Number 458460), is a unit for wild monkey conservation, which occupies an area of 259.54 hectares. It is located in the municipality of Guapimirim (between latitudes 22°27′ and 22°32′ S and longitudes 42°50′ and 42°56′ W), almost in the centre of RJ state, at the *Serra dos Órgãos* slopes, in the Atlantic forest (Fig. 1). The study had the authorization of the Fiocruz Research Ethical Committee. The Fiocruz Ethics Committee for the Use of Laboratory Animals agreed to the protocol for sample collection. The handling of monkeys was exclusively done by CPRJ technicians.

A total of 122 NHPs of the family Callitrichidae were sampled in June 2015 (n = 4), January 2016 (n = 115) and July 2016 (n = 4). The origin of all animals, sex, species and date of sample collection are shown in Additional file 1: Table S1. Of these animals, 10 belonged to the genus *Callithrix* (five Geoffroy marmosets—*Callithrix geoffroyi*, two common marmosets—*Callithrix jacchus*, and three hybrids); 23 of the genus *Saguinus* (nine golden-handed tamarins—*Saguinus midas*, eight pied bare-face tamarins—*S. bicolor*, 3 black-handed tamarins—*Saguinus niger*, one ochraceous bare-face tamarin—*Saguinus martinsi ochraceus*, one Martin’s bare-face tamarin—*Saguinus martinsi martinsi* and one hybrid); 85 of the genus *Leontopithecus* (eight golden lion tamarins—*Leontopithecus rosalia*, four black lion tamarins—*Leontopithecus chrysopygus* and 73 golden-headed lion tamarins—*Leontopithecus chrysomelas*); and five belonged to the genus *Mico* (four black-and-white tassel-ear marmosets—*Mico humeralifer* and one Maués marmoset—*Mico mauesi*) (Additional file 1: Table S1). The species were determined by the veterinaries from CPRJ based on Rylands et al. and Van Roosmalen et al. [26, 27]. Seventy-five NHPs (61%) were captive from conservation centers (CPRJ and Renabra nursery) or Zoos (Belo Horizonte Zoo, Mario Nardelli Zoo, Niteroi Zoo and São Carlos Zoo) and 47 (39%) were wild caught in different areas, including eight in the Brazilian Amazon (Additional file 1: Table S1). It is important to highlight that the wild animals from *Serra da Tiririca* Park in Niteroi (RJ state) were housed in the CPRJ Centre for a short period of time before transfer to be released in conservation areas of Bahia state or other states. No animal was splenectomized at the time of sample collection. Approximately 5 mL of blood were taken in vacuum tubes containing EDTA by femoral vein puncture. Packed red blood cells were separated by centrifugation, frozen and sent to the malaria laboratory at CPqRR, Fiocruz Minas.

### Parasitological and molecular tests

Thick and thin blood smears stained with Giemsa’s solution were examined under 100× optical microscopy for morphological analysis by two expert microscopists using standard methods [37].

DNA extraction from blood samples was performed using the QIAamp DNA mini Kit (Qiagen, Venlo, The Netherlands) according to manufacturer’s recommendations. The DNA was eluted in 45 μL of Buffer AE and stored at −20 °C until it was used. The samples were subjected to nested PCR using primers for identification of human *Plasmodium* species (*P. malariae/P. brasilianum*, *P. vivax/P. simium* and *Plasmodium falciparum*), targeting the small subunit of 18S ribosomal RNA (18S SSU rRNA) gene [38]. Briefly, all PCR reactions were performed in 20 μL final volume containing 250 μM each oligonucleotide primer, 10 μL of master mix (Promega) (0.3 units of Taq Polymerase, 200 μM each deoxyribonucleotide triphosphates and 1.5 mM MgCl_2_) and 1 μL DNA (5–10 ng). For all positive samples the DNA extraction was repeated and PCR performed using 2 μL whenever the use of 1 μL was negative. The PCR assays were performed using an automatic thermocycler (PTC-100TM v.7.0) (MJ Research Inc, USA) and the following cycling parameters were used: an initial denaturation at 95 °C for 5 min followed by 24 cycles of annealing at 58 °C for 2 min, extension at 72 °C for 2 min and denaturation at 94 °C for 1 min followed by a final annealing incubation at 58 °C for 2 min and extension at 72 °C for 5 min. The temperature was then reduced to 4 °C until the samples were removed from the cycler. The second round of PCR was performed with the same parameters of the first reaction, using 1 μL of the amplified DNA from the first reaction and 29 cycles of amplification. It is important to highlight that DNA extraction and master mix preparation were performed in “parasite DNA-free rooms” distinct from each other with different sets of pipettes and using plugged pipette tips to prevent cross-contamination. Every PCR reaction included positive controls for each pair of primers and a negative control with no DNA. The sources of genomic DNA samples that served as positive controls in the nested PCR assays were: i) DNA of *P. brasilianum* of MR4 (Malaria Research and Reference Reagent Resource Center—ATCC, USA); (ii) DNA of patient with high parasitaemia for *P. vivax*; (iii) *P. falciparum* DNA, strain 3D7 maintained in the malaria laboratory (CPqRR-Fiocruz Minas).

The amplified fragments were visualized using electrophoresis on 2% agarose gels in 1× TAE buffer (40 mM Tris–acetate, 1 mM EDTA) with 5 μg/mL ethidium bromide (Invitrogen) in a horizontal system (Bio-Rad) at 100 V for about 30 min. The gels were examined under a UV transilluminator and the image captured under digital system (UVP—Bio-Doc System it). Electrophoresis was performed in a room specifically assigned for analysis of amplified DNA, with appropriate sets of micropipettes and plugged pipette tips.

### Sequencing of PCR-amplified DNA

Two microlitre of PCR products were amplified using 2.0 μM of forward species-specific primer and 1 μL of Big Dye terminator kit for DNA sequencing. The following cycling parameters were used: 96 °C for 1 min, 35 cycles of 96 °C for 15 s, followed by the 58 °C for 15 s and 60 °C for 15 s. The fragments were precipitated using ammonium acetate, suspended in formamide HI-DI (Applied Biosystems) and electrophoretically separated in ABI 3730 DNA automatic sequencer.

### Data analysis

The sequence data were analyzed using the Blast program and the sequences aligned using the Clustal W programs in Bioedit package v7.2.5 [39].

## Results

A total of 122 non-human primates of the family Callitrichidae housed in CPRJ (Fig. 1) were sampled (Additional file 1: Table S1). The NHPs belonged to different species of the genera *Callithrix* (n = 10), *Saguinus* (n = 23), *Leontopithecus* (n = 84) and *Mico* (n = 5) (Table 1). Eight samples (6.6%) were positive by nested PCR for *P. brasilianum/P. malariae*: three *Saguinus* (one *Saguinus martinsi ochraceus*; one *Saguinus martinsi martinsi* and one hybrid), three *Leontopithecus* (one *Leontopithecus rosalia* and two *Leontopithecus chrysomelas*), one *Callithrix* (*Callithrix geoffroyi*) and one *Mico* (*Mico humeralifer*) (Table 1). The highest PCR positivity rates were shown for the genera *Mico* (20%) and *Saguinus* (13%), while a very low frequency of infection was recorded for *Leontopithecus* (3.6%). It is important to highlight that some PCRs were positive only using the double amount of DNA template and in about 25% of samples the PCR showed inconsistent results (sometimes positive, sometimes negative). Negative controls never showed any amplified fragments and positive controls showed fragments with the expected sizes. Importantly, no Giemsa’s solution-stained thick or thin blood smear was positive by optical microscopy.

**Table 1:** *Plasmodium brasilianum* infection in non-human primates of family Callitrichidae

Species	Number of specimens	Positive results by nested PCR	
		Number	Percentage
Genus *Callithrix*			
*C. geoffroyi*	5	1	20%
*C. jacchus*	2	0	0
Hybrid	3	0	0
Total (genus)	10	1	10%
Genus *Leontopithecus*			
*L. chrysomelas*	73	2	2.7%
*L. chrysopygus*	4	0	0
*L. rosalia*	7	1	14%
Total (genus)	84	3	3.6%
Genus *Mico*			
*M. humeralifer*	4	1	25%
*M. mauesi*	1	0	0
Total (genus)	5	1	20%
Genus *Saguinus*			
*S. bicolor*	8	0	0
*S. martinsi martinsi*	1	1	100%
*S. midas*	9	0	0
*S. niger*	3	0	0
*S. martinsi ochraceous*	1	1	100%
Hybrid	1	1	100%
Total (genus)	23	3	13%
Total (family)	122	8	6.6%

**Fig. 2:**
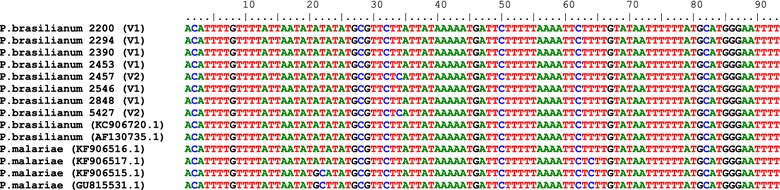
Multiple alignment of *Plasmodium* 18S SSU rRNA gene partial sequences showing two variants (V1 and V2) among Brazilian *P. brasilianum*. The 18S SSU rRNA gene partial sequences (second amplicon from Snounou’s PCR) obtained herein from parasites isolated from Atlantic forest NHPs: *Callitrix geoffroyi* (2294), *Mico humeralifer* (2390), *Leontopithecus chrysomelas* (2453 and 5427), *Leontopithecus rosalia* (2457) and *Saguinus* hybrid (2898); and from Amazonian NHPs: *Saguinus martinsi martinsi* (2200) and *Saguinus martinsi ochraceus* (2546). These sequences were compared to *P. brasilianum* and *P. malariae* sequences from GenBank (accession number)

A species-specific fragment of 18S SSU rRNA from *P. brasilianum* was sequenced to confirm the infection. The sequences obtained were compared to sequences of *P. brasilianum* and *P. malariae* available at Genbank (Fig. 2). A high similarity was shown by alignment between *P. brasilianum* and *P. malariae*. In addition, two distinct variants among *P. brasilianum* were identified.

## Discussion

*Plasmodium brasilianum* infects a large number of species belonging to all families of New World monkeys, in contrast with the majority of other primate-infective *Plasmodium* species, which tend to infect hosts within the same taxonomic family [11, 19]. Within the family Callitrichidae 1193 specimens from 28 species were previously surveyed for *Plasmodium* infection. Only three species of *Saguinus* were naturally infected (*Saguinus niger, Saguinus midas* and *Saguinus geoffroyi*) (Additional file 2: Table S2). Infection of only one *Saguinus geoffroyi* by *P. brasilianum* was described in 1971 [28]. In 1985, the first description of malaria infection in *Saguinus niger* was reported; among 109 Callitrichidae only 4 (3.7%) were found infected by optical microscopy [21]. In 1992, a review of data from five different studies, including 509 Callitrichidae specimens from the genera *Callithrix, Cebuella, Leontopithecus* and *Saguinus* showed an infection rate of 0.8% among this family (4 *Saguinus niger*) also using only optical microscopy [11]. In 2000, one out of 90 *Saguinus midas* infected with *P. brasilianum* by microscopy and 3 out of 54 by PCR were reported in a survey in French Guiana [19]. Here, *P. brasilianum/P. malariae* DNA was identified in two new species of *Saguinus: Saguinus martinsi martinsi* and *Saguinus martinsi ochraceus.* Moreover, samples of other 3 genera: *Callithrix*, *Leontopithecus* and *Mico,* were found positive by PCR for *P. brasilianum/P. malariae* DNA. Therefore, *Plasmodium* DNA was detected by nested PCR in 6.6% of individuals (8/122). Considering the absence of parasite detection in blood smears, the detection limit of the nested PCR protocol used here as <3 parasites/μL blood for human samples, and PCR inconsistencies, it is suggested that the parasitaemia is even lower than expected for infected NHPs [11, 40, 41]. Four out of nine *Plasmodium* infection surveys in Callitrichidae were based only on microscopy [11, 21, 22, 28]. Interestingly, Deane mentioned that Prof. Mauro Barreto had already found *Plasmodium* in one *Callithrix geoffroyi* examined for *Trypanosoma cruzi*, however this information has never been published [11]. Corroborating the findings presented here, splenectomized common marmosets (*Callithrix jacchus*) were experimentally infected with *P. brasilianum* [42–44]. Moreover, the use of a low copy number target in the nested PCR might result in an underestimation of the infection rate, so it needs to be further investigated using more abundant genomic targets.

A previous survey of wild *Leontopithecus chrysomelas* from Niteroi, RJ state (n = 268) based on microscopy and PCR failed to detect *Plasmodium* infection [45]. This survey was conducted during animal transfer from an urban park named *Serra da Tiririca* (Atlantic forest fragment) in Niteroi, to Bahia state (place of natural occurrence for this species of NHP). After capture, the animals were kept in quarantine at CPRJ, where they were examined for *Plasmodium* infection immediately after arrival. Here were examined 31 wild animals from Niteroi that were kept longer at CPRJ and, interestingly, one of them was found infected. This infected animal had arrived at CPRJ in September 2015, and it was examined in January 2016 being positive for *P. brasilianum* infection. None of these animals were surveyed before. Taking into account that 100% of the wild *Leontopithecus chrysomelas* from Niteroi were negative in the previous very large sampling [45], it was suggested that the infected animal had acquired the infection at CPRJ during the 4 months of housing there.

Of the eight animals found infected in this study, two were wild captured in Manaus surroundings (Amazonia state) and transferred to CPRJ more than 6 years ago (*Saguinus martinsi ochraceus* and *Saguinus martinsi martinsi*) (Additional file 1: Table S1). As these animals were never tested before, it is uncertain whether they came infected from Amazonia (where human malaria is endemic in Brazil and where NHP infection is also prevalent) or they became infected at the CPRJ. Our hypothesis is that transmission occurs in the area where the animals are maintained in captivity based on: (i) the majority of infected NHPs were born at CPRJ (Additional file 1: Table S1); (ii) vectors for *Plasmodium* exist in the Atlantic forest area where the CPRJ is located [46–48]; (iii) many potential susceptible NHPs were housed in close proximity to animals found infected [36]; (iv) all positive animals were housed near each other and close to the forest (less than 1 km); (v) stress caused by captivity could result in increased susceptibility to infection and vulnerability to higher parasitaemias, thus favouring transmission [49]; and (vi) autochthonous cases of human malaria have been reported in this municipality [35, 37]. Therefore, in these conditions, infection could spread among NHPs, including species never found infected before as well as human beings. In agreement with this hypothesis, analysis of 18S SSU rRNA gene partial sequences, although of very short length for evolutionary analysis, identified two variants of *P. brasilianum* circulating among NHPs: one shared between Atlantic forest and Amazonian NHPs and a second one identified only in Atlantic forest NHPs.

Our previous identification at CPRJ of new NHP species infected by *P. simium* [36], a much more host restrictive simian parasite than *P. brasilianum* supports the results reported here. Moreover, host specificity of ape malaria parasites could also be affected by captivity, as evidenced by reported transfers of *Plasmodium* across chimpanzees and gorillas in Africa [50].

## Conclusions

Identification of new NHP species infected with *Plasmodium* in captivity suggests that a much large number of potential hosts, such as tamarins and marmosets, could act as malaria reservoirs in the Atlantic forest. However, whether the DNA detected by PCR reflects red blood cells infection, as well as the role of these new potential hosts in malaria transmission in the Atlantic forest need to be further determined, including evaluation of infected vectors. Once autochthonous human cases of malaria are reported in the regions where the monkeys were found infected, the presence of potential wild reservoirs may have important implications for public health by compromising malaria control and eradication efforts. Health governmental agencies need therefore to take into account zoonotic malaria for development of appropriate control strategies in these regions.

## Additional files

**Additional file 1: Table S1.** Non-human primates from the family Callitrichidae housed at the CPRJ.

**Additional file 2: Table S2.** Review of description of natural infection by *Plasmodium brasilianum* in family Callitrichidae.

## Abbreviations

CPRJ: Primate Centre of Rio de Janeiro
NHP: non-human primate
PCR: polymerase chain reaction
18S SSU rRNA: 18S small subunit of ribosomal RNA
