# Supplementary material for: New potential Plasmodium brasilianum hosts: tamarin and marmoset monkeys (family Callitrichidae)
Source: Malar J. 2017 Feb 10;16:71. doi: 10.1186/s12936-017-1724-0 (PMC5303265; doi:10.1186/s12936-017-1724-0)
Supplement: Supplementary file 1 — Additional file 1: Table S1. Non-human primates from the family Callitrichidae housed at the CPRJ. [file 12936_2017_1724_MOESM1_ESM.docx]

**Additional File 1: Table S1 Non-human primates from the family Callitrichidae housed at the CPRJ**

| **Number at CPRJ** | **Species** | **Sex** | **Origin** | **Captive or wild** | **Arrival at CPRJ** | **Date of blood collection** |
| --- | --- | --- | --- | --- | --- | --- |
| 2164 | *Callithrix (hibrid)* | M | São Paulo state | Wild | Nov/2004 | 01/21/16 |
| 2159 | *Callithrix (hibrid)* | F | São Paulo state | Wild | Nov/2004 | 01/21/16 |
| 2521 | *Callithrix (hibrid)* | F | Serra dos Órgãos National Park (RJ state) | Wild | Nov/2008 | 01/21/16 |
| 2268 | *Callithrix geoffroy* | M | Born in CPRJ | Captive | NA | 01/19/16 |
| **2294** | ***Callithrix geoffroyi*** | **M** | **Born in CPRJ** | **Captive** | **NA** | **01/19/16** |
| 3357 | *Callithrix geoffroyi* | M | Monkey from CETAS/RJ | Wild | Sep/2015 | 01/20/16 |
| 3358 | *Callithrix geoffroyi* | M | Monkey from CETAS/RJ | Wild | Sep/2015 | 01/20/16 |
| 3359 | *Callithrix geoffroyi* | F | Monkey from CETAS/RJ | Wild | Sep/2015 | 01/20/16 |
| 2568 | *Callithrix jacchus* | F | Born in CPRJ | Captive | NA | 01/20/16 |
| 2569 | *Callithrix jacchus* | F | Born in CPRJ | Captive | NA | 01/20/16 |
| 1981 | *Leontopithecus chrysomelas* | M | Born in Renabra Nursery (RJ state) | Captive | Sep/2000 | 01/21/16 |
| 1433 | *Leontopithecus chrysomelas* | F | Born in CPRJ | Captive | NA | 01/19/16 |
| 1644 | *Leontopithecus chrysomelas* | M | Born in CPRJ | Captive | NA | 01/21/16 |
| 1859 | *Leontopithecus chrysomelas* | M | Born in CPRJ | Captive | NA | 01/21/16 |
| 1893 | *Leontopithecus chrysomelas* | M | Born in CPRJ | Captive | NA | 01/21/16 |
| 1894 | *Leontopithecus chrysomelas* | M | Born in CPRJ | Captive | NA | 01/21/16 |
| 1982 | *Leontopithecus chrysomelas* | M | Born in CPRJ | Captive | NA | 01/21/16 |
| 2012 | *Leontopithecus chrysomelas* | M | Born in CPRJ | Captive | NA | 01/19/16 |
| 2041 | *Leontopithecus chrysomelas* | F | Born in CPRJ | Captive | NA | 01/20/16 |
| 2122 | *Leontopithecus chrysomelas* | M | Born in CPRJ | Captive | NA | 01/21/16 |
| 2136 | *Leontopithecus chrysomelas* | M | Born in CPRJ | Captive | NA | 01/19/16 |
| 2153 | *Leontopithecus chrysomelas* | F | Born in CPRJ | Captive | NA | 01/19/16 |
| 2186 | *Leontopithecus chrysomelas* | F | Born in CPRJ | Captive | NA | 01/21/16 |
| 2196 | *Leontopithecus chrysomelas* | M | Born in CPRJ | Captive | NA | 01/20/16 |
| 2226 | *Leontopithecus chrysomelas* | M | Born in CPRJ | Captive | NA | 01/20/16 |
| 2228 | *Leontopithecus chrysomelas* | M | Born in CPRJ | Captive | NA | 01/20/16 |
| 2233 | *Leontopithecus chrysomelas* | M | Born in CPRJ | Captive | NA | 01/20/16 |
| 2272 | *Leontopithecus chrysomelas* | M | Born in CPRJ | Captive | NA | 01/19/16 |
| 2308 | *Leontopithecus chrysomelas* | M | Born in CPRJ | Captive | NA | 01/21/16 |
| 2354 | *Leontopithecus chrysomelas* | M | Born in CPRJ | Captive | NA | 01/20/16 |
| 2355 | *Leontopithecus chrysomelas* | F | Born in CPRJ | Captive | NA | 01/20/16 |
| 2364 | *Leontopithecus chrysomelas* | F | Born in CPRJ | Captive | NA | 01/20/16 |
| 2397 | *Leontopithecus chrysomelas* | M | Born in CPRJ | Captive | NA | 01/19/16 |
| 2398 | *Leontopithecus chrysomelas* | M | Born in CPRJ | Captive | NA | 01/21/16 |
| 2445 | *Leontopithecus chrysomelas* | M | Born in CPRJ | Captive | NA | 01/21/16 |
| 2452 | *Leontopithecus chrysomelas* | M | Born in CPRJ | Captive | NA | 01/19/16 |
| **2453** | ***Leontopithecus chrysomelas*** | **F** | **Born in CPRJ** | **Captive** | **NA** | **01/19/16** |
| 2498 | *Leontopithecus chrysomelas* | F | Born in CPRJ | Captive | NA | 01/19/16 |
| 2533 | *Leontopithecus chrysomelas* | F | Born in CPRJ | Captive | NA | 01/21/16 |
| 2548 | *Leontopithecus chrysomelas* | F | Born in CPRJ | Captive | NA | 01/21/16 |
| 2565 | *Leontopithecus chrysomelas* | M | Born in CPRJ | Captive | NA | 01/21/16 |
| 2566 | *Leontopithecus chrysomelas* | M | Born in CPRJ | Captive | NA | 01/21/16 |
| 2570 | *Leontopithecus chrysomelas* | M | Born in CPRJ | Captive | NA | 01/21/16 |
| 2605 | *Leontopithecus chrysomelas* | M | Born in CPRJ | Captive | NA | 01/21/16 |
| 2606 | *Leontopithecus chrysomelas* | M | Born in CPRJ | Captive | NA | 01/21/16 |
| 2607 | *Leontopithecus chrysomelas* | F | Born in CPRJ | Captive | NA | 01/21/16 |
| 2622 | *Leontopithecus chrysomelas* | F | Born in CPRJ | Captive | NA | 01/20/16 |
| 2899 | *Leontopithecus chrysomelas* | M | Born in CPRJ | Captive | NA | 01/21/16 |
| 2900 | *Leontopithecus chrysomelas* | F | Born in CPRJ | Captive | NA | 01/21/16 |
| 1918 | *Leontopithecus chrysomelas* | M | Born in Mário Nardelli Zoo (RJ state) | Captive | Aug/1999 | 01/20/16 |
| 2172 | *Leontopithecus chrysomelas* | M | IBAMA apprehension (RJ state) | Wild | Dec/2004 | 01/19/16 |
| 2173 | *Leontopithecus chrysomelas* | F | IBAMA apprehension (RJ state) | Wild | Dec/2004 | 01/20/16 |
| 3109 | *Leontopithecus chrysomelas* | F | Born in CPRJ | Wild | NA | 01/21/16 |
| 3110 | *Leontopithecus chrysomelas* | F | Born in CPRJ | Wild | NA | 01/21/16 |
| 5391 | *Leontopithecus chrysomelas* | F | Serra da Tiririca Park in Niteroi (RJ state)* | Wild | Dec/2015 | 01/22/16 |
| 5392 | *Leontopithecus chrysomelas* | F | Serra da Tiririca Park in Niteroi (RJ state)* | Wild | Dec/2015 | 01/22/16 |
| 5393 | *Leontopithecus chrysomelas* | M | Serra da Tiririca Park in Niteroi (RJ state)* | Wild | Dec/2015 | 01/22/16 |
| 5394 | *Leontopithecus chrysomelas* | M | Serra da Tiririca Park in Niteroi (RJ state)* | Wild | Dec/2015 | 01/22/16 |
| 5395 | *Leontopithecus chrysomelas* | M | Serra da Tiririca Park in Niteroi (RJ state)* | Wild | Dec/2015 | 01/22/16 |
| 5396 | *Leontopithecus chrysomelas* | M | Serra da Tiririca Park in Niteroi (RJ state)* | Wild | Dec/2015 | 01/22/16 |
| 5397 | *Leontopithecus chrysomelas* | F | Serra da Tiririca Park in Niteroi (RJ state)* | Wild | Dec/2015 | 01/22/16 |
| 5399 | *Leontopithecus chrysomelas* | M | Serra da Tiririca Park in Niteroi (RJ state)* | Wild | Dec/2015 | 01/22/16 |
| 5400 | *Leontopithecus chrysomelas* | F | Serra da Tiririca Park in Niteroi (RJ state)* | Wild | Dec/2015 | 01/22/16 |
| 5402 | *Leontopithecus chrysomelas* | M | Serra da Tiririca Park in Niteroi (RJ state)* | Wild | Dec/2015 | 01/22/16 |
| 5403 | *Leontopithecus chrysomelas* | M | Serra da Tiririca Park in Niteroi (RJ state)* | Wild | Dec/2015 | 01/22/16 |
| 5404 | *Leontopithecus chrysomelas* | F | Serra da Tiririca Park in Niteroi (RJ state)* | Wild | Dec/2015 | 01/22/16 |
| 5405 | *Leontopithecus chrysomelas* | M | Serra da Tiririca Park in Niteroi (RJ state)* | Wild | Dec/2015 | 01/22/16 |
| 5406 | *Leontopithecus chrysomelas* | M | Serra da Tiririca Park in Niteroi (RJ state)* | Wild | Nov/2015 | 01/22/16 |
| 5408 | *Leontopithecus chrysomelas* | F | Serra da Tiririca Park in Niteroi (RJ state)* | Wild | Nov/2015 | 01/22/16 |
| 5409 | *Leontopithecus chrysomelas* | F | Serra da Tiririca Park in Niteroi (RJ state)* | Wild | Nov/2015 | 01/22/16 |
| 5410 | *Leontopithecus chrysomelas* | M | Serra da Tiririca Park in Niteroi (RJ state)* | Wild | Nov/2015 | 01/22/16 |
| 5415 | *Leontopithecus chrysomelas* | M | Serra da Tiririca Park in Niteroi (RJ state)* | Wild | Nov/2015 | 01/22/16 |
| 5424 | *Leontopithecus chrysomelas* | M | Serra da Tiririca Park in Niteroi (RJ state)* | Wild | Sep/2015 | 01/20/16 |
| **5427** | ***Leontopithecus chrysomelas*** | **F** | **Serra da Tiririca Park in Niteroi (RJ state)*** | **Wild** | **Sep/2015** | **01/20/16** |
| 5429 | *Leontopithecus chrysomelas* | F | Serra da Tiririca Park in Niteroi (RJ state)* | Wild | Sep/2015 | 01/20/16 |
| 5430 | *Leontopithecus chrysomelas* | M | Serra da Tiririca Park in Niteroi (RJ state)* | Wild | Sep/2015 | 01/20/16 |
| 5438 | *Leontopithecus chrysomelas* | M | Serra da Tiririca Park in Niteroi (RJ state)* | Wild | Sep/2015 | 01/20/16 |
| 5440 | *Leontopithecus chrysomelas* | F | Serra da Tiririca Park in Niteroi (RJ state)* | Wild | Oct/2015 | 01/22/16 |
| 5441 | *Leontopithecus chrysomelas* | F | Serra da Tiririca Park in Niteroi (RJ state)* | Wild | Oct/2015 | 01/22/16 |
| 5444 | *Leontopithecus chrysomelas* | M | Serra da Tiririca Park in Niteroi (RJ state)* | Wild | Oct/2015 | 01/22/16 |
| 5445 | *Leontopithecus chrysomelas* | M | Serra da Tiririca Park in Niteroi (RJ state)* | Wild | Oct/2015 | 01/22/16 |
| 5446 | *Leontopithecus chrysomelas* | M | Serra da Tiririca Park in Niteroi (RJ state)* | Wild | Oct/2015 | 01/22/16 |
| 5447 | *Leontopithecus chrysomelas* | M | Serra da Tiririca Park in Niteroi (RJ state)* | Wild | Oct/2015 | 01/22/16 |
| *1821* | *Leontopithecus chrysopygus* | M | Born in CPRJ | Captive | NA | 02/06/15 |
| *2571* | *Leontopithecus chrysopygus* | M | Born in CPRJ | Captive | NA | 02/06/15 |
| *2109* | *Leontopithecus chrysopygus* | F | Born in CPRJ | Captive | NA | 02/06/15 |
| *2572* | *Leontopithecus chrysopygus* | F | Born in CPRJ | Captive | NA | 02/06/15 |
| 3503 | *Leontopithecus rosalia* | F | Born in Belo Horizonte Zoo (Minas Gerais state) | Captive | Nov/2015 | 07/07/16 |
| 2393 | *Leontopithecus rosalia* | M | Born in CPRJ | Captive | NA | 01/20/16 |
| 2394 | *Leontopithecus rosalia* | F | Born in CPRJ | Captive | NA | 01/20/16 |
| 2435 | *Leontopithecus rosalia* | M | Born in CPRJ | Captive | NA | 07/07/16 |
| **2457** | ***Leontopithecus rosalia*** | **M** | **Born in CPRJ** | **Captive** | **NA** | **01/20/16** |
| 2256 | *Leontopithecus rosalia* | M | Born in Niteroi Zoo (RJ state) | Captive | May/2006 | 01/20/16 and 07/07/2016 |
| 2352 | *Leontopithecus rosalia* | M | Born in CPRJ | Captive | NA | 07/07/16 |
| **2390** | ***Mico humeralifer*** | **M** | **Born in CPRJ** | **Captive** | **NA** | **01/20/16** |
| 2391 | *Mico humeralifer* | F | Born in CPRJ | Captive | NA | 01/20/16 |
| 2599 | *Mico humeralifer* | F | Born in CPRJ | Captive | NA | 01/21/16 |
| 1856 | *Mico humeralifer* | F | Born in CPRJ | Captive | NA | 01/20/16 |
| 2765 | *Mico mauesi* | M | CETAS Manaus (Amazonas state) | Wild | May/2013 | 01/21/16 |
| **2898** | ***Saguinus* (hibrid)** | **M** | **Born in CPRJ** | **Captive** | **NA** | **01/19/16** |
| 1684 | *Saguinus bicolor* | F | Rescued from Amazônia (AM) | Wild | Sep/1996 | 01/19/16 |
| 2418 | *Saguinus bicolor* | M | Born in CPRJ | Captive | NA | 01/21/16 |
| 2560 | *Saguinus bicolor* | M | Born in CPRJ | Captive | NA | 01/20/16 |
| 2588 | *Saguinus bicolor* | M | Born in CPRJ | Captive | NA | 01/21/16 |
| 2601 | *Saguinus bicolor* | F | Born in CPRJ | Captive | NA | 01/21/16 |
| 3041 | *Saguinus bicolor* | F | Born in CPRJ | Captive | NA | 01/19/16 |
| 2766 | *Saguinus bicolor* | F | CETAS Manaus (Amazonas state) | Wild | May/2013 | 01/20/16 |
| 3479 | *Saguinus bicolor* | F | CETAS Manaus (Amazonas state) | Wild | Nov/2015 | 01/19/16 |
| **2200** | ***Saguinus martinsi martinsi*** | **F** | **CETAS Manaus (Amazonas state)** | **Wild** | **Mar/2015** | **01/19/16** |
| **2546** | ***Saguinus martinsi ochraceus*** | **M** | **CETAS Manaus (Amazonas state)** | **Wild** | **Dec/2010** | **01/19/16** |
| 2300 | *Saguinus midas* | M | Born in CPRJ | Captive | NA | 01/19/16 |
| 2301 | *Saguinus midas* | M | Born in CPRJ | Captive | NA | 01/20/16 |
| 2349 | *Saguinus midas* | F | Born in CPRJ | Captive | NA | 01/20/16 |
| 2540 | *Saguinus midas* | F | Born in CPRJ | Captive | NA | 01/20/16 |
| 2562 | *Saguinus midas* | M | Born in CPRJ | Captive | NA | 01/20/16 |
| 2611 | *Saguinus midas* | M | Born in CPRJ | Captive | NA | 01/20/16 |
| 2801 | *Saguinus midas* | M | Born in CPRJ | Captive | NA | 01/21/16 |
| 2579 | *Saguinus midas* | F | CETAS Manaus (Amazonas state) | Wild | Dec/2011 | 01/20/16 |
| 2280 | *Saguinus midas* | F | São Carlos Zoo (São Paulo state) | Captive | Nov/2006 | 01/20/16 |
| 2309 | *Saguinus niger* | M | Born in CPRJ | Captive | NA | 01/20/16 |
| 2395 | *Saguinus niger* | M | Born in CPRJ | Captive | NA | 01/20/16 |
| 2580 | *Saguinus niger* | M | CETAS Manaus (Amazonas state) | Wild | Dec/2011 | 01/21/16 |
| *Animals transferred from urban Atlantic Forest (Serra da Tiririca Park) in Niteroi (RJ) to other areas | | | |  |  |  |
| CETAS - Center for apprehension of wild animals (illegal traphicking, rescue, etc) | | | |  |  |  |
| IBAMA - Brazilian Institute of Environment and renewable natural resources | | | |  |  |  |
| Bold - Positive NHPs in PCR for *Plasmodium brasilianum/P.malariae* | | | |  |  |  |
