# Supplementary material for: New potential Plasmodium brasilianum hosts: tamarin and marmoset monkeys (family Callitrichidae)
Source: Malar J. 2017 Feb 10;16:71. doi: 10.1186/s12936-017-1724-0 (PMC5303265; doi:10.1186/s12936-017-1724-0)
Supplement: Supplementary file 2 — Additional file 2: Table S2. Review of description of natural infection by Plasmodium brasilianum in family Callitrichidae. [file 12936_2017_1724_MOESM2_ESM.docx]

| **Additional File 2: Review of description of natural infection by *Plasmodium brasilianum* in family Callitrichidae** | | | | | | |
| --- | --- | --- | --- | --- | --- | --- |
| **Species^a^** | **Actual nomenclature^b^** | **Popular name** | **Origin^c^** | **Specimens** | **Positives** | **Reference** |
|  |  |  |  |  |  |  |
| **Genus *Callibella*** |  |  |  |  |  |  |
| *C. humilis* | *C. humilis* (Van Roosmalen et al., 1998) | Black-crowned dwarf marmoset | captive (RO) | 1 | 0 | Araújo, 2013 |
| **Genus *Callithrix*** |  |  |  |  |  |  |
| *C. aurita coelestis* | *C. aurita* (É. Geoffroy in Humoldt, 1812) | Buffy-tufted-ear marmoset | Wild | 6 | 0^d^ | Deane, 1992^e^ |
| *C. argentate emiliae* | *Mico argentatus* (Linnaeus, 1966) | Silvery marmoset | Wild | 4 | 0^d^ | Deane, 1992^e^ |
| *C. emiliae* | *Mico emiliae* (Thomas, 1820) | Snethlage´s marmoset | Wild (RO) | 4 | 0^d^ | Lourenço-de-Oliveira, 1995 |
| *C. geoffroyi* | *C. geoffroyi* (É. Geoffroy in Humoldt, 1812) | Geoffroy´s tufted-ear marmoset | Wild | 30 | 0^d^ | Deane, 1992^e^ |
| *C. geoffroyi* | *C. geoffroyi* (É. Geoffroy in Humoldt, 1812) | Geoffroy´s tufted-ear marmoset | # | 5 | **1** | Herein |
| *C. humeralifer* | *Mico humeralifer* (É. Geoffroy in Humboldt, 1812) | Black and white tassel-ear marmoset | Wild | 2 | 0^d^ | Deane, 1992^e^ |
| *C. jacchus* | *C. jacchus* (Linnaeus, 1758) | Common marmoset | Wild | 67 | 0^d^ | Deane, 1992^e^ |
| *C. jacchus* | *C. jacchus* (Linnaeus, 1758) | Common marmoset | Wild (GO) | 5 | 0 | Duarte, 2008 |
| *C. jacchus* | *C. jacchus* (Linnaeus, 1758) | Common marmoset | # | 2 | 0 | Herein |
| *C. penicillata jordani* | *C. penicillata* (É. Geoffroy, 1812) | Black-tuffed-ear marmoset | Wild | 141 | 0^d^ | Deane, 1992^e^ |
| *Callitrhrix spp.* | *Callithrix spp.* | Atlantic forest marmoset | Wild (SP) | 39 | 0 | Duarte, 2008 |
| Hybrid | NA | NA | # | 3 | 0 | Herein |
| ***Genus Cebuella*** |  |  |  |  |  |  |
| *C.pygmaea* | *C. pygmaea pygmaea* (Spix, 1823) | Western pygmy marmoset | Wild | 1 | 0^d^ | Deane, 1992^e^ |
| *C.pygmaea* | *C. pygmaea pygmaea* (Spix, 1823) | Western pygmy marmoset | Wild (RO) | 4 | 0 | Araújo, 2013 |
| **Genus *Leontopithecus*** |  |  |  |  |  |  |
| *L.chrysomelas* | *L.chrysomelas* (Kuhl, 1820) | Golden-headed lion tamarin | Wild (RJ) | 268 | 0 | Aitken, 2016 |
| *L.chrysomelas* | *L.chrysomelas* (Kuhl, 1820) | Golden-headed lion tamarin | # | 73 | **2** | Herein |
| *L. chrysopygus* | *L. chrysopygus* (Mikan, 1823) | Black lion tamarin | # | 4 | 0 | Herein |
| *L. rosalia* | *L. rosalia* (Linnaeus, 1766) | *golden lion tamarin* | Wild | 28 | 0^d^ | Deane, 1992^e^ |
| *L. rosalia* | *L. rosalia* (Linnaeus, 1766) | *golden lion tamarin* | # | 7 | **1** | Herein |
| **Genus *Mico*** |  |  |  |  |  |  |
| *M. humeralifer* | *Mico humeralifer* (É. Geoffroy in Humboldt, 1812) | Black-and-white tassel-ear marmoset, Santarém marmoset | # | 4 | **1** | Herein |
| *M. mauesi* | *M. mauesi* (Mittermeier et al., 1992) | Maués marmoset | # | 1 | 0 | Herein |
| *M. melanurus* | *M. melanurus* (É. Geoffroy in Humboldt, 1812) | Black-tailed marmoset | Wild (RO) | 3 | 0 | Araújo, 2013 |
| **Genus *Saguinus*** |  |  |  |  |  |  |
| *S. bicolor* | *S. bicolor* (Spix, 1823) | Brazilian bare-face tamarin, pied tamarin, bare-face tamarin | Wild | 12 | 0^d^ | Deane, 1992^e^ |
| *S. bicolor* | *S. bicolor* (Spix, 1823) | Brazilian bare-face tamarin, pied bare-face tamarin | # | 8 | 0 | Herein |
| *S. fuscicollis* | *S. fuscicollis fuscicollis* (Spix, 1823) | Spix´s saddle-back tamarin | Wild | 26 | 0^d^ | Deane, 1992^e^ |
| *S. fuscicollis mura* | *S. fuscicollis mura* (Rohe et al., 2009) | Grey-fronted saddle-back tamarin | Wild (AM) | 2 | 0 | Bueno, 2013 |
| *S. geoffroyi* | *S. geoffroyi* (Pucheran, 1845) | Geoffroy´s tamarin, titi marmoset | Wild (Panamá) |  | 1 | Baerg 1971 |
| *S. imperator* | *S. imperator imperator* (Goeldi, 1907) | Black-chinned emperor tamarin | Wild | 2 | 0^d^ | Deane, 1992^e^ |
| *S. labiatus labiatus* | *S. labiatus labiatus (É. Geoffroy in Humboldt, 1812)* | Geoffroy´s red-bellied tamarin | Wild | 2 | 0^d^ | Deane, 1992^e^ |
| *S. labiatus* | *S. labiatus labiatus (É. Geoffroy in Humboldt, 1812)* | Geoffroy´s red-bellied tamarin | Wild (RO) | 1 | 0 | Araújo, 2013 |
| *S. labiatus grisoevertex* | *S. labiatus labiatus* (É. Geoffroy in Humboldt, 1812)^g^ | Geoffroy´s red-bellied tamarin | Wild | 5 | 0^d^ | Deane, 1992^e^ |
| *S. labiatus rufiventer* | *S. labiatus rufiventer* (Gray, 1843) | Gray´s red-bellied tamarin | Wild (AM) | 1 | 0 | Bueno, 2013 |
| *S. martinsi martinsi* | *S. martinsi martinsi* (Thomas, 1912) | Martins´ bare-face tamarin | # | 1 | **1** | Herein |
| *S. martinsi ochraceus* | *S. martinsi ochraceus* (Hershkovitz, 1966) | Ochraceous bare-face tamarin | # | 1 | **1** | Herein |
| *S. midas midas* | *S. midas* (Linnaeus, 1758) | Midas tamarin, golden-handed tamarin | Wild | 2 | 0^d^ | Deane, 1992^e^ |
| *S. midas midas* | *S. midas* (Linnaeus, 1758) | Midas tamarin, golden-handed tamarin | Wild (AM) | 10 | 0^d^ | Lourenço-de-Oliveira, 1995 |
| *S. midas* | *S. midas* (Linnaeus, 1758) | Midas tamarin, golden-handed tamarin | Wild (French Guiana) | 90 | **3^e^** | Fandeur, 2000 |
| *S. midas* | *S. midas* (Linnaeus, 1758) | Midas tamarin, golden-handed tamarin | # | 9 | 0 | Herein |
| *S. midas niger* | *S. niger* (É. Geoffroy, 1803) | Western black-handed tamarin | Wild | 109 | **4^d^** | Arruda et al. 1985 |
| *S. midas niger* | *S. niger (É. Geoffroy, 1803)* | Western black-handed tamarin | Wild | 178 | **4^d^** | Deane, 1992^e^ |
| *S. niger* | *S. niger (É. Geoffroy, 1803)* | Western black-handed tamarin | # | 3 | 0 | Herein |
| *S. mystax* | *S. mystax mystax* (Spix, 1823) | Spix´s mustached tamarin | Wild | 3 | 0^d^ | Deane, 1992^e^ |
| *S. weddelli* | *Saguinus weddelli weddelli* (Deville, 1849) | Weddell´s saddle-back tamarin | Wild (RO) | 6 | 0 | Araújo, 2013 |
| *S. fuscicollis weddelli* | *Saguinus weddelli weddelli* (Deville, 1849) | Weddell´s saddle-back tamarin | Wild (RO) | 19 | 0^d^ | Lourenço-de-Oliveira, 1995 |
| Hybrid | NA | NA | # | 1 | **1** | Herein |
| Total |  |  |  | 1193 | 19 |  |
| a Scientific name used in the original reference of Plasmodium survey | | | | | |  |
| b Nomenclature according to Rylands et al. 2009, 2012, 2016, Anthony et al. 2000 | | | | | |  |
| c Brazilian state abbreviatures: AM - Amazonas, GO - Goias, RO - Rondonia, RJ - Rio de Janeiro, SP - São Paulo | | | | | | |
| d based only on opitical microscopy | | | | | |  |
| e Deane 1992 - review of the data published in Deane, 1976, Arruda, 1985, Lourenço-de-oliveira, 1988, 1990, Deane et al. 1989 | | | | | |  |
| f Two positive out of 90 by MO and 3 out 54 by PCR | | | | | |  |
| g Actual correspondent cientific name not accurated | | | | | |  |
| # See Additional File 1 | | | | | |  |
| NA - not applicable, | | | | | |  |
